# Orthogonal targeting of osteoclasts and myeloma cells for radionuclide stimulated dynamic therapy induces multidimensional cell death pathways

**DOI:** 10.7150/thno.60757

**Published:** 2021-06-22

**Authors:** Alexander Zheleznyak, Matthew Mixdorf, Lynne Marsala, Julie Prior, Xiaoxia Yang, Grace Cui, Baogang Xu, Steven Fletcher, Francesca Fontana, Gregory Lanza, Samuel Achilefu

**Affiliations:** 1 Department of Radiology, Washington University School of Medicine, St. Louis, MO 63110, USA.; 2 Department of Medicine, Washington University School of Medicine, St. Louis, MO 63110, USA.; 3 Department of Pharmaceutical Sciences, University of Maryland School of Pharmacy, Baltimore, MD 21201, USA.; 4 Department of Biomedical Engineering, Washington University, St. Louis, MO 63105, USA.; 5 Department of Biochemistry and Molecular Biophysics, Washington University School of Medicine, St. Louis, MO 63110, USA.

**Keywords:** multiple myeloma, bone marrow, tumor microenvironment, orthogonal drug delivery, nanomicelles, photosensitizer, Cerenkov radiation

## Abstract

**Rationale:** Multiple myeloma (MM) is a multifocal malignancy of bone marrow plasma cells, characterized by vicious cycles of remission and relapse that eventually culminate in death. The disease remains mostly incurable largely due to the complex interactions between the bone microenvironment (BME) and MM cells (MMC). In the “vicious cycle” of bone disease, abnormal activation of osteoclasts (OCs) by MMC causes severe osteolysis, promotes immune evasion, and stimulates the growth of MMC. Disrupting these cancer-stroma interactions would enhance treatment response.

**Methods:** To disrupt this cycle, we orthogonally targeted nanomicelles (NM) loaded with non-therapeutic doses of a photosensitizer, titanocene (TC), to VLA-4 (α4ß1, CD49d/CD29) expressing MMC (MM1.S) and αvß3 (CD51/CD61) expressing OC. Concurrently, a non-lethal dose of a radiopharmaceutical, ^18^F-fluorodeoxyglucose ([^18^F]FDG) administered systemically interacted with TC (radionuclide stimulated therapy, RaST) to generate cytotoxic reactive oxygen species (ROS). The *in vitro* and *in vivo* effects of RaST were characterized in MM1.S cell line, as well as in xenograft and isograft MM animal models.

**Results:** Our data revealed that RaST induced non-enzymatic hydroperoxidation of cellular lipids culminating in mitochondrial dysfunction, DNA fragmentation, and caspase-dependent apoptosis of MMC using VLA-4 avid TC-NMs. RaST upregulated the expression of BAX, Bcl-2, and p53, highlighting the induction of apoptosis via the BAK-independent pathway. The enhancement of multicopper oxidase enzyme F5 expression, which inhibits lipid hydroperoxidation and Fenton reaction, was not sufficient to overcome RaST-induced increase in the accumulation of irreversible function-perturbing α,ß-aldehydes that exerted significant and long-lasting damage to both DNA and proteins. *In vivo,* either VLA-4-TC-NM or αvß3-TC-NMs RaST induced a significant therapeutic effect on immunocompromised but not immunocompetent MM-bearing mouse models. Combined treatment with both VLA-4-TC-NM and αvß3-TC-NMs synergistically inhibited osteolysis, reduced tumor burden, and prevented rapid relapse in both *in vivo* models of MM.

**Conclusions:** By targeting MM and bone cells simultaneously, combination RaST suppressed MM disease progression through a multi-prong action on the vicious cycle of bone cancer. Instead of using the standard multidrug approach, our work reveals a unique photophysical treatment paradigm that uses nontoxic doses of a single light-sensitive drug directed orthogonally to cancer and bone cells, followed by radionuclide-stimulated generation of ROS to inhibit tumor progression and minimize osteolysis in both immunocompetent murine and immunocompromised human MM models.

## Introduction

MM is neoplasia of plasma cells in the bone marrow and is the second most common hematological malignancy 1-7. Clinically, the disease progresses from asymptomatic monoclonal gammopathy of undetermined significance (MGUS) and smoldering multiple myeloma (SMM) to an increase in the number of malignant plasma cells and the appearance of CRAB symptoms (hypercalcemia, renal failure, anemia, and bone lesions) 8-10. At the pathogenetic level, several mutations can contribute to the clonal expansion of malignant plasma cells 7. However, advanced MM and MGUS are often indistinguishable in the load and type of karyotypic and sequence abnormalities 11, suggesting a key role of MM cell-extrinsic factors in determining tumorigenic progression of bone lesions 12-15. Indeed, several lines of evidence show that some pro-MM changes in BME accompany, or even precede, the appearance of bone lesions 16-19.

Bone marrow (BM), the primary anatomical site of hematopoiesis 1, 20-22, comprises diverse cell populations 23. Particularly, hematopoietic cells localized in the BM interact extensively with the bone marrow stromal cells (BMSC) and cells that regulate bone morphology, including matrix-deposing osteocytes and osteoblasts of mesenchymal origin and bone-resorption osteoclasts (OCs) of myeloid lineage 24. Together with the extracellular matrix proteins (ECM) 25-28, bone resident cells interact with MM cells to promote tumor growth, survival, immune suppression, and resistance to chemotherapy 17, 19, 29-31. These complex interactions result in osteolytic lesions, which occur in 90% of MM patients 32 with a high rate of skeletal-related events that affect morbidity, quality of life, and survival 33.

The extensive heterogeneity and high frequency of MM relapse require autologous stem cell transplantation for eligible patients and the administration of combination drugs that include immunomodulatory drugs (thalidomide, lenalidomide, pomalidomide), corticosteroids (dexamethasone), proteasome inhibitors (bortezomib, carfilzomib, ixazomib), deacetylase inhibitors (panobinostat), and monoclonal antibodies (Elotuzumab, Daratumumab) 34-42. To prevent or minimize severe skeletal-related events, current therapies include inhibitors of bone resorption (zoledronic acid, pamidronates, denosumab) and radiation 32. These interventions are used at sufficiently high doses to exert a therapeutic effect but also induce dose-limiting side effects. A strategy to overcome this challenge is to use sub-lethal doses of drugs and selectively enhance therapeutic effect in the target cells.

MM and the BME provide a rich source of biomarkers for targeting diverse cells, including CD38, CS1, VLA-4, and αvß3 proteins. Previously, we demonstrated that VLA-4 integrin is upregulated on MM cells and MM protective lymphocytes and macrophages 43, thus constituting a useful therapeutic target. To minimize organ and systemic toxicity, we developed a new treatment paradigm called Cerenkov radiation-induced therapy where light-sensitive drugs (photosensitizers) can absorb Cerenkov radiation from radiopharmaceuticals 42, 43 to generate cytotoxic ROS. We recently coined the term radionuclide stimulated therapy (RaST) to account for other potential contributing factors to the observed therapeutic effects other than Cerenkov radiation 44. The ability of RaST to induce therapeutic effect only when the two components co-localize in the same or adjacent cells enables us to administer nontoxic amounts of both photosensitizers and radiopharmaceuticals to effect precision therapy. Using contact-facilitated drug delivery strategy 45, 46, we recently demonstrated the high loading capacity and delivery of unmodified photosensitizers in NMs targeted to tumors 43. Compared to large nanoparticles with limited diffusivity 47-49, the small size of the NMs (20 nm) facilitates the penetration and distribution in the highly vascularized and compartmentalized bone marrow where MM resides 50. Despite the improvement in therapeutic response, the rate of MM relapse remained high, probably caused by either cell-intrinsic adaptations or protective interactions with the bone niche. Previous studies established that increased osteoclastogenesis and subsequent osteolytic activity are essential for MM survival 6, 51. OC differentiation and activity promote MM progression and relapse by stimulating proliferation and reactivation from dormancy of MMC 52. The integrin αvß3, which is over-expressed on these cells but not in MM cells, plays a vital role in the osteolytic function of OCs 53, 54. A unique benefit of RaST is that the photophysical ROS-generating mechanism is applicable in any targeted cell type, favoring the use of the same light-sensitive drug delivered orthogonally to targeted cells to achieve disparate multicellular therapy.

**Figure 1:**
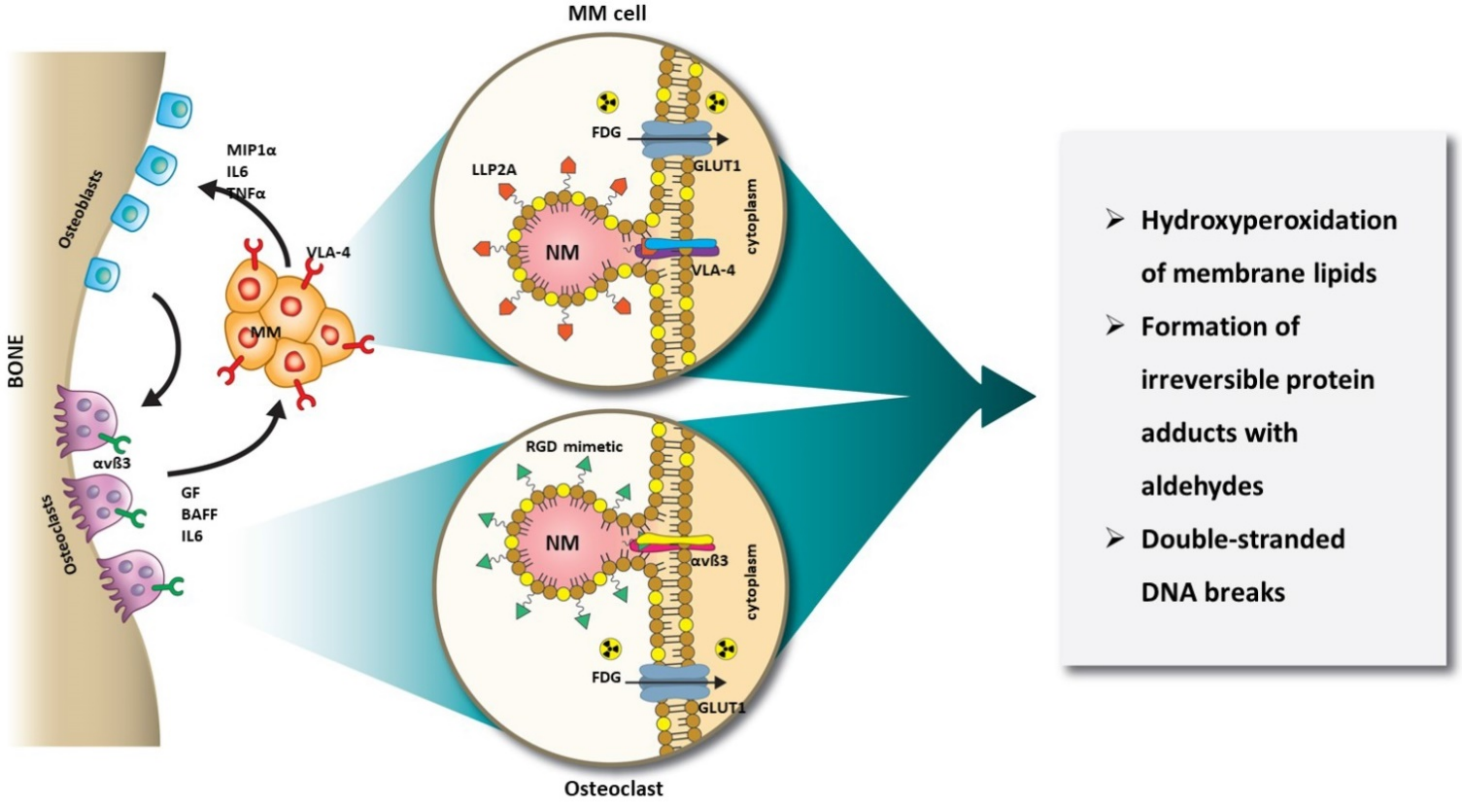
** Schematic illustration of orthogonal RaST.** NMs are loaded with a light-sensitive drug, TC, but directed to different cells that express VLA-4 (MM) and αvß3 (OCs) in the BM. The expression of GLUT1 on both MM and OCs provides additional selectivity for eradicating only cells that express GLUT1 and either VLA-4 or αvß3.

In this study, we harnessed the orthogonal expression of VLA-4 on the cell surface of MM 55, 56, and αvß3 on the plasma membrane of OCs 57, 58, as well as the expression of GLUT1 (CD71) glucose transporter on both cell types 59-62 for a new intercellular RaST. Incorporation of the photosensitizer, TC, into NMs (20 nm) targeted to either αvß3 or VLA-4 allowed us to deliver TC selectively to OC and MM cells, respectively. Using [^18^F]FDG to initiate ROS generation, we elucidated the mechanism of RaST-induced cell death (Figure 1). Our results point to a paradigm where the direct depletion of pro-tumorigenic cells in the BME combines with a multidimensional cell death mechanism to achieve a sustainable therapeutic effect in a disease that currently remains incurable. By using the same drug to treat both cell types, we mitigated the cumulative toxic effects of multidrug therapy on healthy tissue. A requirement for treating MM patients with relapsed disease is to add at least a drug from a non-refractory group. With many pharmacologic drugs unable to meet this threshold, multicellular RaST is a potential standalone therapy or a component of multidrug therapy of MM and other bone lesions.

## Methods

### Cell lines and reagents

MM1.S human MM 63 and 5TGM1 murine MM 64 cell lines, either naïve or carrying Click Beetle Red (CBR) luciferase and Green Fluorescent Protein (GFP) reporters (MM1.S/CBR/GFP and 5TGM/CBR/GFP, respectively), as well as MM1.S/CBR/GFP resistant to RaST and MM1.S/CBR/GFP without CD49d, were generously provided by Dr. DiPersio (Washington University School of Medicine, WUSM, St. Louis). Cells were routinely cultured in complete medium (CM) consisting of Iscove's Modified Dulbecco's Media (Thermo Fisher Scientific, Waltham, MA) supplemented with 10% heat-inactivated fetal bovine serum (FBS, ThermoFisher Scientific, Waltham, MA) and 50 µg/mL Gentamycin (Thermo Fisher Scientific, Waltham, MA). The cells were routinely washed in phosphate-buffered saline (PBS, Thermo Fisher Scientific, Waltham, MA), pH 7.4. VLA-4 and αvß3 targeted NM were loaded with TC (Bis(cyclopentadienyl)titanium(IV) dichloride, Sigma-Aldrich, St. Louis, MO) 43, 46. Protein content was routinely measured with Pierce^TM^ bicinchoninic acid (BCA) assay (Thermo Fisher Scientific Waltham, MA).

### Synthesis of VLA4-PEG_2000_-PE

A VLA-4 antagonist based on the highly selective LLP2A 65 peptidomimetic, which binds selectively to activated α4ß1 heterodimers 65 was synthesized and coupled to polyethylene glycol-phosphatidyethanolamine anchor. Briefly, rink amide 4-methylbenzhydrylamine resin (MBHA) resin was fluorenylmethyloxycarbonyl chloride (Fmoc) deprotected with 20% piperidine in dimethylformamide (DMF). Fmoc-Ach-OH dissolved in hydroxy-benzotriazole (HOBt) and 1,3-diisopropylcarbodiimide (DIC) in DMF was coupled at RT for 2 h. The Fmoc deprotection with 20% piperidine, and serial coupling and deprotection cycles with Fmoc-Aad(tBu) and Fmoc- Lys(Dde) were performed. After removal of Fmoc, a solution of 2-(4-(3-o-tolylureido)phenyl)acetic acid, HOBt and DIC in DMF was added overnight, washed, and the Dde protecting group removed with 2% hydrazine in DMF. A solution of trans-3-(3-pyridyl) acrylic acid, HOBt and DIC in DMF was added and coupling was ensured via a negative Kaiser test. The crude product was cleaved with 95% trifluoroacetate (TFA): 2.5% water: 2.5% triisopropylsilane, precipitated with diethyl ether and purified using RP-HPLC. VLA-4 ligand dissolved in ethanol was mixed with 2-iminothiolane in methanol and allowed to react for 2 h @ 25 ºC. N-(4-(P-maleimidophenyl) butyrl)-phosphatidylethanolamine (MPB-PEG_2000_-DSPE) was added and incubated for 2 h. The purified lyophilized sample was a white solid (VLA-4-PEG-PE, Calculated, 3781; Observed, m/z. 3780).

### Synthesis of αvß3-integrin antagonist

The αvß3-integrin antagonist was a quinalone nonpeptide developed by Bristol-Myers Squibb Medical Imaging (US patent 6,511,648 and related patents), which was initially reported and characterized as the ^111^In-DOTA conjugate RP478 and cyan 5.5 homologue TA145 66. The specificity of the αvß3 ligand mirrors that of LM609 and has a 15-fold preference for the Mn^2+^ activated receptor (21 nM) 67. The IC_50_ estimates for αvß5_,_ α5ß1 and GP IIbIIIa were > 10 µM (BMSMI, Billerica, MA, USA, US patent 6,511,648 and related patents). The antagonist amide coupled to PEG_2000_-phosphatidylethanolamine (αvß3-PEG-PE) was a gift from Kereos, Inc., St. Louis, MO. (Calculated: 4021, Observed: m/z 4020).

### Synthesis of VLA-4-TC-NM and αvß3-TC-NM

Targeted titanocene nanomicelles were prepared as previously described 43. Briefly, phospholipid/polysorbate 80 micelles were prepared as a microfluidized suspension comprised of 20% polysorbate tween 80 (v/v) (NOF America, White Plains, NY), a 2.0% (w/v) surfactant commixture, and 1.7% (w/v) glycerin in filtered MilliQ Nanopure water. The surfactant commixture included 0 or 2 mole% titanocene dichloride (TiCl_2_, Sigma-Aldrich, St. Louis, MO) and 0 or 0.15 mole% of a VLA4-PEG-PE or αvβ3-PEG-PE with the remainder phosphatidylcholine (>98% purity, NOF America, White Plains, NY). The surfactant components were dried from organic solvent into a film, resuspended in nanopure water, and combined with polysorbate 80, buffer, Nanopure water, and glycerin. The mixture was sonicated at 4 °C for 3 min, then microfluidized (LV1, Microfluidics, Westwood, MA) at 20,000 psi for 5 passes. Dynamic light scattering (DLS) physicochemical characteristics (nominal of 10 estimates) of the αvß3-NM were: particle size: 18 ± 2 nm, polydispersity: 0.2 ± 0.02, zeta potential 1.4 ± 0.6 mV and for the VLA-4-NM were: particle size: 20 ± 1.4 nm, polydispersity: 0.3 ± 0.01, zeta potential: -0.3± 0.2 mV. The NM were filtered with 0.2 µm filter into sterile serum vials, preserved under inert gas, capped and crimp-sealed, then stored at 4 °C until use. Further data schematically illustrating the NM and TEM, as well as the titanocene NM pharmacokinetic and biodistribution were previously described 43.

### [^18^F]FDG production

[^18^F]FDG was produced with an average specific activity 70-299 mCi/mL by the Mallinckrodt Institute of Radiology's Cyclotron Facility and Nuclear Pharmacy at Washington University School of Medicine in compliance with current good manufacturing practices.

### Murine osteoclast differentiation

Bone marrow macrophages (BMM) were isolated and induced to differentiate into OCs according to previously methodology 68, with modifications. Fox Chase SCID beige or C57BL/6-KaLwRij male mice (5-7 weeks old) were euthanized, and femurs and tibiae were aseptically dissected. Both ends of bones were removed, and bone marrow was extracted using centrifugation at 10,000 × g for 10 s. The pellet was suspended in Roswell Park Memorial Institute 1640 medium (RPMI-1640) supplemented with 10% heat-inactivated HyClone defined Fetal Bovine Serum (Thermo Fisher Scientific, Waltham, MA) and 50 µg/mL Gentamicin (RPMI-10G) containing 5 × 10^-6^ mg/mL recombinant mouse M-CSF (R&D Systems, Minneapolis, MN) and plated on 10 cm Petri dishes (Valmark, Thomas Scientific, Swedesboro, NJ). The plating density of BMM from 2 mice for each plate was routinely maintained. The plates were incubated overnight at 37 °C and humidified 5% CO_2_ atmosphere. The non-adherent cells were harvested, washed once with RPMI-10G, and plated on 24-well tissue culture plates (TPP, Midwest Scientific, Valley Park, MO) at 4 × 10^5^ cells per well in RPMI-10G supplemented with 100 ng/mL RANKL (R&D Systems, Minneapolis, MN). The medium was renewed every 72 h for a minimum of seven days.

### Cell viability

MTS (3-(4,5-dimethylthiazol-2-yl)-5-(3-carboxymethoxyphenyl)-2-(4-sulfophenyl)-2H-tetrazolium) assay, a colorimetric assay for assessing cell viability, was performed using CellTiter 96^®^ Aqueous One Solution Cell Proliferation Reagent (Promega, Madison, MI). After treatment, the CM was carefully removed from each well and replaced with 0.5 mL of PBS containing 50 µL tetrazolium compound (3-(4,5-dimethylthiazol-2-yl)-5-(3-carbooxymethoxyphenyl)-2-(4-sulfophenyl)-2H-tetrazolium, inner salt; MTS) and phenazine ethosulfate (PES, electron coupling reagent) (CellTiter 96^®^ Aqueous One Solution Cell Proliferation Reagent, Promega, Madison, WI) to determine the percentage of live cells. The plates were incubated at 37 °C for 1-4 h to allow the tetrazolium compound to develop. The resulting absorbance was detected at 490 nm with a Synergy/NEO2 multi-mode reader (BioTek, Winooski, VT).

### *In vitro* RaST

*In vitro* RaST experiments were performed in 24 well plates containing 4 x 10^5^ cells per well in 1 mL of CM. The treatment was initiated by the addition of either VLA-4-TC-NM (5 µL per well, 0.5 µg TC) or αvß3-TC-NM (5 µL per well, 0.5 µg TC), or their combination. The NM were allowed to bind to target receptors at 4 °C for 1 h. The medium with unbound NM was carefully removed and replaced with 1 mL fresh medium. At this time, 3.7 MBq/mL (0.1 mCi) [^18^F]FDG in saline was added to the appropriate wells, and the plates were placed in the 37 °C/5% CO_2_ humidified atmosphere for the indicated amounts of time. After the completion of treatment, the medium was carefully removed and 0.5 mL of PBS containing 50 µL MTS reagent (CellTiter 96^®^ Aqueous One Solution Cell Proliferation Reagent, Promega, Madison, WI) was added to each well to determine the percentage of live cells. The plates were incubated at 37 °C for 1-4 h to allow the tetrazolium compound to be reduced to form formazan dye. The resulting absorbance was detected at 490 nm with a Synergy/NEO2 multi-mode reader (BioTek, Winooski, VT). The data were reported as a percent of live cells compared to the untreated cells (100%).

### Reactive oxygen species assay

TC is a photosensitizer and is known to generate ROS upon irradiation by UV light or Cerenkov radiation 69, 70. ROS measurements were carried out at RT using 2',7'-dichlorodihydrofluorescein diacetate (H_2_DCFDA, Thermo Fisher Scientific, Waltham, MA) as ROS indicator. RaST treatments were performed as described in the *in vitro* RaST section. After the VLA-4-TC-NM RaST and the controls were added to the appropriate wells, H_2_DCFDA was added to each well as follows. H_2_DCFDA was reconstituted in DMSO immediately prior to being added to each well at 5 µM final concentration and the plates were incubated in humidified atmosphere at 37 °C/5% CO_2_ for 72 h. At the end of the incubation period, the dye was excited at 495 nm and the emitted fluorescence was detected at 520 nm with a Synergy/NEO2 multi-mode reader (BioTek, Winooski, VT). The cells were mechanically harvested with a rubber-tipped cell scraper (Sarstedt, Newton, NC), washed twice with PBS, lysed with 200 µL RIPA buffer at 4 °C for 30 min and the total protein content was determined with the BCA assay. The data were reported as fluorescence intensity per µg of protein.

### Oxidative stress markers profile

Three oxidative stress markers were included in the matabolomic analysis of MM1.S cells: 3β,5α,6β-trihydroxycholestane (triol, CAS 1253-84-5), 7-ketocholesterol (CAS 566-28-9), and malondialdehyde (MDA, CAS 542-78-9). To investigate the oxidative stress markers resulting from RaST, the cells were treated with VLA-4-TC-NM RaST or left untreated as in the *in vitro* RaST section. At the end of treatment, the cells were mechanically harvested with a rubber-tipped cell scraper (Sarstedt, Newton, NC) and washed twice with PBS. The cultured cell samples were lysed with 200 μL of 4:1 MeOH/Water, containing 10 ng each of deuterated triol-d7, deuterated 7-ketocholesterol-d7, deuterated 4-hydroxy nonenal-d_3_ (4-HNE-d3), and deuterated 4-hydroxy hexenal-d_3_ (4-HHE-d3) as internal standards. An aliquot (50 μL) of the cell lysate was dried under a stream of nitrogen. The sterol markers were derivatized with dimethylglycine (DMG), while the aldehyde markers in the second aliquot (50 μL) were derivatized with O-benzylhydroxylamine to improve MS sensitivity as well as chromatographic properties. The analysis was performed using a Shimadzu 20AD HPLC system coupled to a tandem mass spectrometer (API-6500+Qtrap: Applied Biosystems, Waltham, MS) operated in multiple reaction monitoring mode (MRMM). The positive ion spray ionization mode (ESIM) was used for detection of analytes and the internal standards. The samples were injected in triplicate for data averaging. Data processing was conducted with Analyst 1.6.3 software (Applied Biosystems, Waltham, MS). For each analyte, the peak area was calculated and compared to the peak area of the corresponding internal standard. The resulting ratios for each analyte were reported as relative abundance.

### Lipid hydroperoxide assay

Lipid hydroperoxide measurements were performed using the lipid hydroperoxide test (Northwest Life Sciences Specialties, LLC, Vancouver, WA). Intracellular lipid peroxides oxidize ferrous iron (Fe^2+^) to form ferric iron (Fe^3+^). The amount of Fe^3+^ reflects the lipid peroxide content and is detected by its reaction with 3,3'-Bis[N,N-bis(carboxymethyl)aminomethyl]-o-cresolsulonephtalein (Xylenol Orange). MM1.S cells were either treated with VLA-4-TC-NM RaST, VLA-4-TC-NM alone, [^18^F]FDG alone or left untreated for 72 h. The cells were then mechanically harvested, lysed, 10 µL lysate aliquots from each well were reserved for the protein content measurement, and treated with catalase to inactivate endogenous H_2_O_2_. The lysates were reacted with the ferrous iron reagent. The resulting Fe^3+^ was detected with Xylenol Orange as a chromogen at 560 nm using Synergy/NEO2 multi-mode reader (BioTek, Winooski, VT). The data was normalized to protein content of each sample measured with Pierce^TM^ BCA protein assay kit (Thermo Fisher Scientific, Waltham, MA) and reported as OD per µg of protein.

### Caspase-3 assay

Caspase 3 levels were determined with Apo-ONE^®^ Homogeneous Caspase-3/7 Assay kit (Promega, Madison, WI). MM1.S cells were plated in 24 well plates at 4 × 10^5^ per well in CM and either left untreated or treated with VLA-4-TC-NM RaST, VLA-4-TC-NM alone, and [^18^F]FDG alone for 72 h as described in the *in vitro* RaST section. Each condition was tested in triplicate. At the end of treatment, the CM was replaced with 250 µL PBS and 250 µL Apo-ONE^®^ Homogeneous Caspase-3/7 reagent rhodamine 110, (bis-(N-CBZ-L-aspartyl-L-glutamyl-L-valyl-L-aspartic acid amide; Z-DEVD-R110) was added to the wells. The intracellular active Caspase 3/7 removed the DEVD peptides and released the strongly fluorescent R110, which was then excited at 499 nm, and the resulting fluorescence was detected at 521 nm with a Synergy/NEO2 multi-mode reader (BioTek, Winooski, VT). Fluorescence intensities from VLA-4-TC-NM RaST, VLA-4-TC-NM alone, and [^18^F]FDG alone were compared to the fluorescence intensity obtained from the untreated cells and expressed as a ratio over the untreated cells.

### Flow cytometry

DNA fragmentation, such as double-strand breaks, and the associated upregulation of the DNA repair mechanisms is one of the downstream effects of intracellular oxidative stress damage. The degree of DNA repair process was assessed by measuring the level of γH2AX, a phosphorylated histone variant 71. MM1.S cells were treated with VLA-4-TC-NM RaST, VLA-4-TC-NM, [^18^F]FDG or left untreated for 72 h as described in the *in vitro* RaST section. At the end of the incubation period, the cells were mechanically harvested, and washed three times with FACS buffer (PBS supplemented with 0.1% FBS). After the cells were permeabilized with BD Phosflow™ Perm III buffer (BD Biosciences, San Jose, CA), the staining was performed with mouse anti-human γH2AX PE-pS139, clone N1-431 (BD Biosciences, San Jose, CA), 0.5 µg/10^6^ cells for 60 min at 4 °C. Concurrently, the cell cycle phases of cells that have undergone treatment and the controls were determined with the 4′,6-diamidino-2-phenylindole stain (DAPI, BD Biosciences, San Jose, CA) at 8 µg/10^6^ cells. The dead cells were identified and quantified using the membrane-impermeable dye Propidium iodide staining solution (PI, BD Biosciences, San Jose, CA). The expression levels of CD49d, CD61, CD71, and GLUT1 were determined using the following antibodies: PE mouse anti-human CD49d clone 9F10, FITC mouse anti-human CD51/CD61 clone 23C6, PE mouse anti-human CD71 clone M-A712, Alexa Fluor 647 mouse anti-human GLUT1 (all from BD Biosciences, San Jose, CA). The stained cells were analyzed with the Gallios flow cytometer (Beckman Coulter Life Sciences, Brea, CA) and the resulting data were quantified with FlowJo software (Ashland, OR).

### Ferroptosis inhibition with Ferrostatin-1

Ferroptosis inhibitor 3-amino-4-(cyclohexylamino)-benzoic acid, ethyl ester (FST-1, Cayman Chemical, Ann Arbor, MI) was used to investigate the involvement of Ferroptosis in RaST-mediated cell death. MM1.S were plated and either left untreated or treated with VLA-4-TC-NM RaST, as well as VLA-4 and FDG controls, as described in the *in vitro* RaST section. FST-1 (10 µM) was added concomitantly with the VLA-4-TC-NM RaST treatment. After 72 h incubation at 37 °C/5% CO_2_ humidified atmosphere, the CM was carefully removed from each well and replaced with 0.5 mL of PBS containing 50 µL tetrazolium compound (CellTiter 96® Aqueous One Solution Cell Proliferation Reagent, Promega, Madison, WI) to determine the percentage of live cells. The plates were incubated at 37 °C for 1-4 h to allow the tetrazolium compound to develop. The resulting absorbance was detected at 490 nm with a Synergy/NEO2 multi-mode reader (BioTek, Winooski, VT).

### Western immunoblot assay

MM1.S cells were treated with VLA-4-TC-NM RaST, VLA-4-TC-NM, [^18^F]FDG or left untreated for 72 h as described in the *in vitro* RaST section. Following treatment, the cells were mechanically dissociated from 24-well plates using rubber-tipped cell scrapers (Sarstedt, Newton, NC) and washed 3 times with PBS. Cells were then incubated with 25 mM Tris-HCl pH 7.6, 150 mM NaCl, 1% NP-40, 1% sodium deoxycholate, 0.1% SDS buffer (RIPA lysis buffer, Abcam, Cambridge, MA) supplemented with EDTA-free protease inhibitor cocktail (Pierce, ThermoFisher, Waltham, MA) for 60 min at 4 °C followed by centrifugation at 12,000 x g for 10 min. Protein content was quantified using the BCA protein assay (Pierce, ThermoFisher Scientific, Waltham, MA). Lysates (50 µg total protein) were denatured for 5 min at 100 °C and proteins were separated by sodium dodecyl sulfate polyacrylamide gel electrophoresis (SDS-PAGE) at 100 V for 1.5 h using Mini-PROTEAN TG precast gels with Tris/Glycine/SDS buffer system (BioRad, Hercules, CA). The proteins were transferred to a polyvinylidene fluoride membrane at 50 V for 2 h (PVDF, Millipore, Burlington, MA). After blocking for 1 h in TBST blocking buffer (10 mM Tris-HCl, 150 mM NaCl, and 0.05% [v/v] Tween-20, pH 7.5, 5% (w/v) non-fat milk), the PVDF membrane was rinsed once with TBST and incubated overnight at 4 °C with 1° antibody diluted in TBST blocking buffer. After two washes, 5 min each, the membrane was incubated with 2° antibodies conjugated with horseradish peroxidase (HRP) for 1 h at room temperature. The unbound secondary antibody was removed with 3 washes in TBST, 10 min each. Bound 2° antibodies were detected using SuperSignal^TM^ West Pico Plus Detection Substrate (Thermo Fischer Scientific, Waltham, MA) and ChemiDoc^TM^ Imager (BioRad, Hercules, CA). The following 1° antibodies were used for immunoblotting: CD61 (1:1000, unconjugated, clone SJ19-09, Novus Biologicals, Centennial, CO); GLUT1 (1:1000, unconjugated, clone D3J3A, Cell Signaling Technology, Danvers, MA); Bak (1:1000, unconjugated, #3814, Cell Signaling Technology, Danvers, MA); Bax (1:1000, unconjugated, #2772, Cell Signaling Technology, Danvers, MA); p53 (1: 1000, unconjugated, clone 1C12, Cell Signaling Technology, Danvers, MA); Bcl-2 (1:1000, unconjugated, clone D17C4, Cell Signaling Technology, Danvers, MA); α-actin (1:1000, unconjugated, #4967, Cell Signaling Technology, Danvers, MA). The following 2° antibodies were used for immunoblotting: anti-rabbit IgG-HRP (1:10000, conjugated to HRP, sc-2357, Santa Cruz Biotechnology, Dallas, TX).

### Mass Spectrometry-based Label-free Protein Quantification

The MM1.S cells were treated with VLA-4-TC-NM RaST or left untreated as stated in the *in vitro* RaST section. Each condition was performed in triplicate. At the end of treatment, the cells were mechanically harvested with a rubber-tipped cell scraper (Sarstedt, Newton, NC) and washed twice with PBS. Samples were subjected to reduction for 10 min (10 mM TCEP/50 mM ammonium bicarbonate pH 8/100 µL) and alkylation for 30 min (25 mM Iodoacetamide), both at room temperature, followed by digestion with 0.5 µg trypsin (SOLu-Trypsin Dimethylated, Sigma) at 37 °C overnight. The digest was acidified to the final 1% TFA before cleaning up with Pierce C18 tip (Thermo Fisher Scientific, San Jose, CA) following the vendor's instruction. The extracted peptides were dried and resuspended prior to LC-MS/MS analysis.

LC-MS/MS analysis was carried out on an Orbitrap Fusion Lumos (Thermo Fisher Scientific, San Jose, CA) mass spectrometer coupled with a U3000 RSLCnano HPLC (Thermo Fisher Scientific, San Jose, CA). The peptide separation was carried out on a 75 µm × 50 cm PepMap C18 column (Thermo Fisher Scientific, San Jose, CA) at a flow rate of 0.3 μL/min and the following gradient: time = 0-4 min, 2% B isocratic; 4-8 min, 2-10% B; 8-83 min, 10-25% B; 83-97 min, 25-50% B; 97-105 min, 50-98% B. Mobile phase consisted of A: 0.1% formic acid; mobile phase B: 0.1% formic acid in acetonitrile. The instrument was operated in the data-dependent acquisition mode in which each MS1 scan was followed by higher-energy collisional dissociation (HCD) of as many precursor ions in 2-second cycle (Top Speed method). The mass range for the MS1 was done using the FTMS was 365 to 1800 m/z with resolving power set to 60,000 at 400 m/z and the automatic gain control (AGC) target set to 1,000,000 ions with a maximum fill time of 100 ms. The selected precursors were fragmented in the ion trap using an isolation window of 1.5 m/z, an AGC target value of 10,000 ions, a maximum fill time of 100 ms, a normalized collision energy of 35 ms, and an activation time of 30 ms. Dynamic exclusion was performed with a repeat count of 1, exclusion duration of 30 s, and a minimum MS ion count for triggering MS/MS set to 5000 counts.

Sequence mapping and label-free quantification were done using Proteome Discoverer (version PD 2.3). Database searches were established against the Human reference proteome (Uniprot.org) with the Byonic search engine launched by PD. The digestion enzyme was trypsin. Oxidation of methionine and acetylation of N-terminal of protein were specified as variable modifications. Protein quantification was achieved by using total intensities of all precursors. Pathways discoveries were performed using the Kyoto Encyclopedia of Genes and Genomes (KEGG), the WiKiPathways database, and the REACTOME Pathway database.

### Histology

Femurs from treated and control animals were excised at predetermined time points and immediately placed in 10% neutral buffered formalin solution (VWR, West Chester, PA), where they remained for 48 h. The femurs were rinsed six times with PBS for 15 min each and decalcified with 14% EDTA for two weeks. After decalcification, the femurs were rinsed six times with PBS for 15 min each. The femurs were hydrated consecutively with 30%, 50%, and 70% alcohol for 15 min each. The femurs were embedded in paraffin, and 5 µm sections were obtained. The sections were stained with Hematoxylin/Eosin (H&E) and the Tartrate Resistant Acid Phosphatase (TRAP) stains by the Washington University Musculoskeletal Research Core. The stained sections were imaged with the Zeiss Axio Scan.Z1 (Zeiss-USA, San Diego, CA), and the images were analyzed with the ZEN 2.3 software (Zeiss-USA, San Diego, CA). Positive TRAP stain cells from the entire section were counted and presented as OC per section.

### Animal models

All animal experiments were conducted in compliance with Washington University School of Medicine (WUSM) Institutional Animal Care and Use Committee (IACUC) guidelines and the Guide for the Care and Use of Laboratory Animals. Female Fox Chase SCID beige mice (5-6 weeks old) were obtained from Charles River Laboratory (Wilmington, MA). C57BL/6-KaLwRij mice predisposed to MM 72, 73 were obtained from Dr. Greg Mundy (OsteoScreen, Inc., San Antonio, TX) and maintained at WUSM animal facility. Animals purchased from vendors were allowed to acclimate for 1 week prior to studies and all animals were provided with food and water *ad libitum.* When needed, euthanasia was performed by cervical dislocation after anesthesia with 5% isoflurane (Pivetal®, DCM, WUSM). Prior to cell implantation, each cell line was tested for the reporter expression by performing *in vitro* BLI with 1 x 10^5^ cells plated in a black 96-well plate. MM1.S cells (1 x 10^6^ per mouse in 100 µL saline) were injected intravenously (tail vein) in Fox Chase SCID beige mice for the xenograft MM model. 5TGM cells (1 x 10^6^ per mouse in 100 µL saline) were injected intravenously (tail vein) in C57BL/6-KaLwRij mice for the isograft MM model. Tumor progression was monitored by detection of live cells with BLI.

### Bioluminescent imaging (BLI)

*In vivo* bioluminescence imaging of animals bearing MM1.S/CBR/GFP and 5TGM/CBR/GFP cells was performed on the days indicated with an IVIS Lumina (PerkinElmer, Waltham, MA; Living Image 3.2, 1-300 sec exposures, binning 2-8, FOV 12.5cm, f/stop 1, and open filter). Mice were injected intraperitoneally with 150 mg/kg D-luciferin in PBS (Gold Biotechnology, St. Louis, MO) and imaged 10 min later under isoflurane anesthesia (2%, vaporized in O_2_). Total photon flux (radiance, photons/sec/cm^2^/steradian) was measured from fixed region of interest (ROI) over the entire ventral side of the mice using Living Image 2.6. A precipitous drop in photon flux was considered to be an indication of defective circulation and the affected animal was removed from the observation group.

### *In vivo* RaST

*In vivo* RaST was performed in disseminated xenograft and isograft models. Animals (n = 5 per group) were systemically implanted with 1 × 10^6^ of either MM1.S/CBR/GFP (5-6 weeks old male Fox Chase SCID beige mice) or 5TGM/CBR/GFP (5-6 weeks old male C57BL/6-KaLwRij mice) cells through the tail vein. Six days after implantation, tumor progression was determined using BLI (baseline). Animals with a whole-body flux of about 1 x 10^6^ were fasted for 16 h and treated with αvß3-TC-NM + VLA-4-TC-NM RaST, αvß3-TC-NM RaST, VLA-4-TC-NM RaST, or left untreated. Each αvß3-TC-NM RaST and VLA-4-TC-NM RaST treatment consisted of 50 µL NM (5 µg TC) mixed 1:1 with saline to minimize viscosity. The αvß3-TC-NM + VLA-4-TC-NM RaST treatment consisted of 50 µL (5 µg TC) of each NM type mixed with 50 µL saline. [^18^F]FDG (29.6 MBq/animal) was administered intraperitoneally 60-90 min later. The therapy was repeated once weekly for 5 weeks and monitored with weekly BLI.

### Data analysis and statistics

*In vitro* RaST experiments were repeated three times with each condition performed in triplicate to control for the intra-experimental variability. The experiments were not randomized and the analysis was not blind. Metabolomics and proteomics studies were conducted using triplicate samples for each condition. *In vivo* RaST sample sizes were determined using previous experience 74. Specifically, the sample size would depend on the effect size as defined by the mean difference between untreated and treated groups. For example, two-sided t-test would predict a sample size of n = 5 with 80% power to detect an effect size of 2.1 with a type I error rate of 0.05. Quantification of OC in bone sections was performed using both femurs from n = 5 animals per condition. Data and statistical analyses were performed using GraphPad Prism 9.0.1 (151) (GraphPad Software, Inc., La Jolla, CA) and Microsoft Excel. For data with two groups and one variable, a two-tailed unpaired *t*-test was used. Data with one variable and multiple groups were analyzed with a one-way ANOVA and Tukey's or Dunnett's multiple comparisons test to determine the adjusted P-value. Data with two variables and multiple groups were analyzed with a two-way ANOVA and Tukey's multiple comparisons test to determine the adjusted P-value.

The data were expressed as mean ± SD, unless indicated otherwise. Differences at the 95% confidence level (P < 0.05) were considered to be statistically significant.

## Results

### Convergence of nontoxic doses of TC and [^18^F]FDG in VLA-4 (MM) expressing cells selectively inhibits tumor proliferation

**Figure 2:**
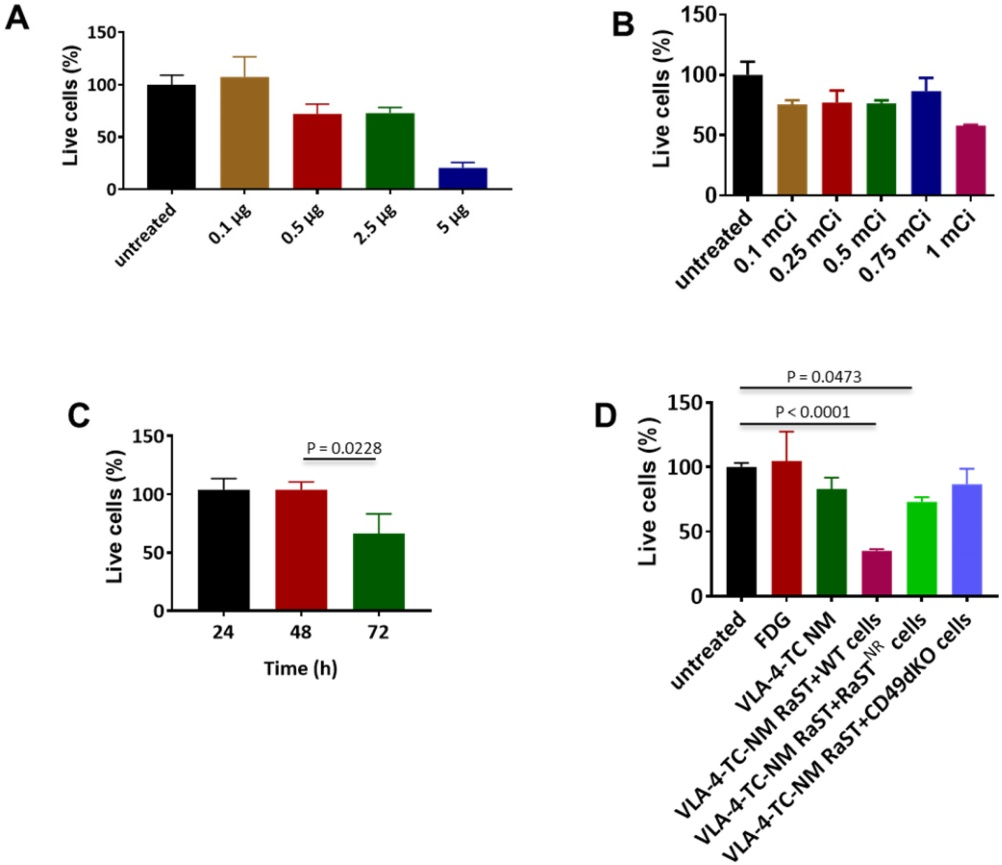
** Non-lethal amounts of TC and [^18^F]FDG converge in target cells to induce cell death**. **(A)** VLA-4 targeted NM loaded with 0.1 µg TC did not lead to cell death; Quantitative data are shown as percent of live cells compared to the untreated cells. **(B)** [^18^F]FDG administered at 3.7 MBq-27.75 MBq (0.1 mCi-0.75 mCi) induce moderate degree of cell death; **(C)** VLA-4-TC-NM RaST induced about 40% MM1.S cell death 72 h after treatment; statistical significance was determined using unpaired two-tailed t-test and a 95% confidence level. The difference between 48 h and 72 h VLA-4-TC-NM RaST was significant (P = 0.0228). **(D)** VLA-4-TC-NM RaST-induced cell death requires the expression of VLA-4 by MM1.S cells; MM1.S cells expressing VLA-4 (WT) were compared to VLA-4-TC-NM RaST-resistant MM1.S cells (NR) expressing low levels of VLA-4 and VLA-4 knockout cells (CD49dKO). The data were analyzed with one-way ANOVA and Dunnett's multiple comparisons tests. Untreated versus RaST + WT: P < 0.0001; untreated versus VLA-4-TC-NM RaST + RaST-resistant (RaST^NR^) cells: P = 0.0473.

Conjugation of drugs to biological carriers for delivery to different cell types presents several challenges, including modifying the drug's intrinsic properties, pharmacokinetics, and intracellular distribution that could disrupt drug action. To circumvent these hurdles, we loaded the same amount of non-modified TC inside unilamellar phospholipid NMs using the method we reported previously 75. A small-molecule peptidomimetic (LLP2A) that targets activated VLA-4 43 or a quinolone molecule that has a high affinity for activated αvß3 49 were inserted onto the surface of the NMs to obtain VLA-4-TC-NMs and αvß3-TC-NMs, respectively. Using the VLA-4-expressing human myeloma cell line, MM1.S 65, we determined the optimal non-therapeutic doses for both TC and [^18^F]FDG to prevent intrinsic toxicity by the RaST component at high doses. Treatment of MM1.S cells with increasing amounts of VLA-4-TC-NMs resulted in dose-dependent cell death after incubating for 24 h. While 0.5 µg, 2.5 µg, and 5 µg TC killed about 25%, 25%, and 75% of the MM cells, respectively, treatment with 0.1 µg TC produced no appreciable cell death (Figure 2A). Similarly, [^18^F]FDG killed less than 20% of the cells at <1 mCi after 24 h (Figure 2B). Based on these results, we chose the lowest amount of TC (0.1 µg) and [^18^F]FDG (0.1 mCi) for subsequent *in vitro* RaST studies.

A delayed cytotoxic effect of RaST was observed at 72 h after treating MM1.S cells with the nontoxic amounts of VLA-4-TC-NM and [^18^F]FDG (Figure 2C). Using the 72 h time point, we demonstrated that the response of MM1.S cells to VLA-4-TC-NM RaST required the co-localization of both TC and [^18^F]FDG to exert a sustainable cell-killing effect (Figure 2D). By contrast, RaST was ineffective in killing RaST-resistant MM1.S cells which have low VLA-4 expression 43 or genetically modified VLA-4 knockout MM1.S cells 43 (Figure 2D). Taken together, these data demonstrate a synergistic interaction between nontoxic doses of a photosensitizer and a radionuclide capable of emitting Cerenkov radiation to exert specific and durable therapeutic effects on cancer cells.

### VLA-4-TC-NM RaST increases ROS and non-enzymatic lipid hydroperoxidation in MM1.S cells

**Figure 3:**
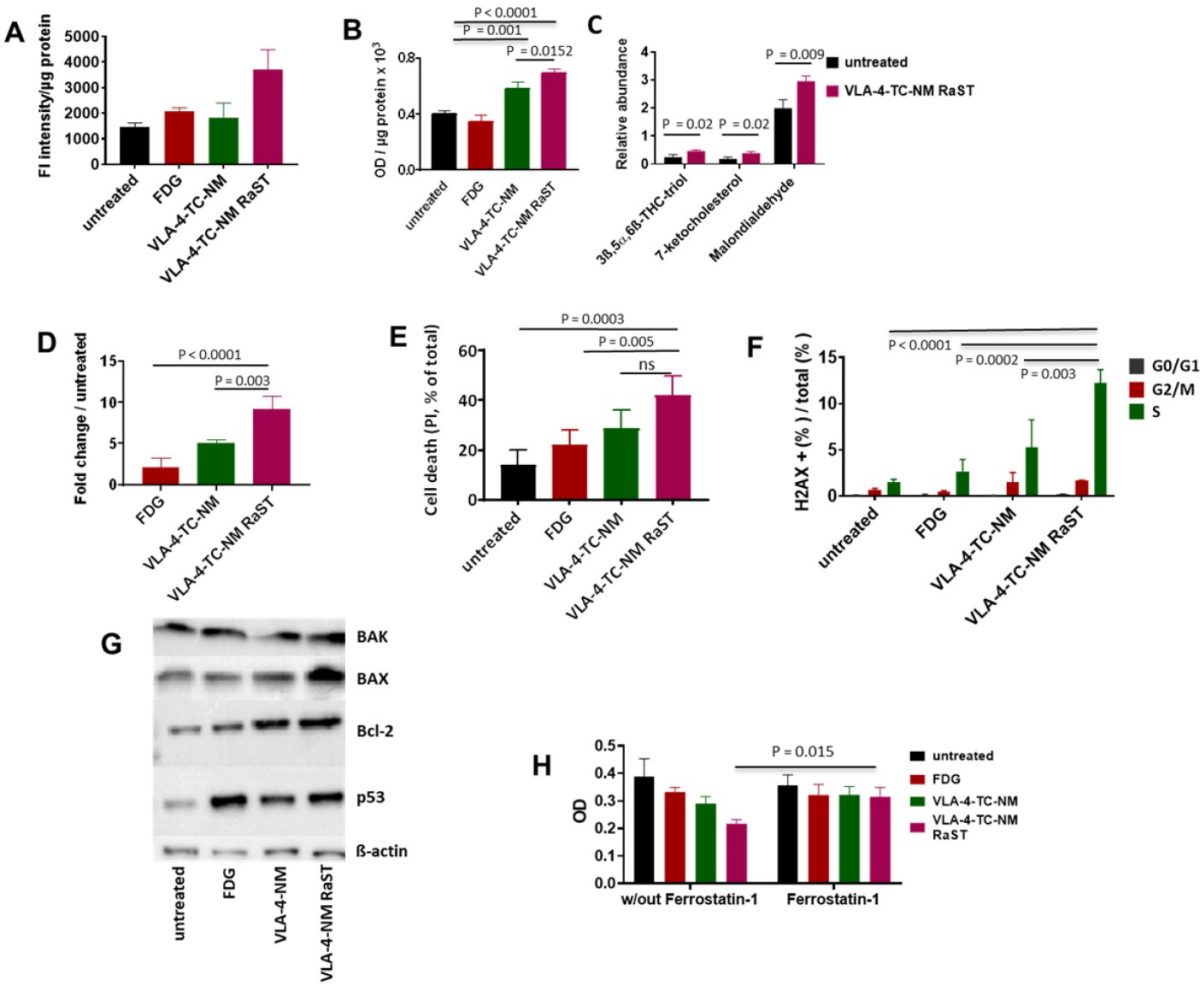
** Mechanism of VLA-4-TC-NM RaST. (A)** VLA-4-TC-NM RaST generates significantly more ROS (P=0.0039) compared to either [^18^F]FDG (NS) or VLA-4-TC-NM alone (NS) when compared to no treatment; 4 x 10^5^ MM1.S cells were treated with [^18^F]FDG, VLA-4-TC-NM, VLA-4-TC RaST, or left untreated. ROS measurements were performed 72 h later using H_2_DCFDA. **(B)** Hydroperoxydation of PUFA increased in cells treated with VLA-4-TC-NM RaST. MM1.S cells were plated and treated as in **(A).** The level of PUFA hydroperoxidation was measured with a lipid peroxidation assay 72 h later. VLA-4-TC-NM and VLA-4-TC-NM RaST produced significantly more lipid hydroperoxidation (P = 0.001 and P < 0.0001, respectively) than no treatment control.** (C)** VLA-4-TC-NM RaST-induced PUFA hydroperoxidation generated reactive aldehydes 72 h after administration. The cells were treated with VLA-4-TC-NM RaST or left untreated. 3ß,5α,6ß-THC-triol, 7-ketocholesterol, and malondialdehyde levels were significantly higher (P = 0.02, P = 0.02, P = 0.009, respectively) in treated cells; multiple t-tests were used for the statistical analysis. **(D)** Caspase-3 level was significantly higher in cells treated with VLA-4-TC-NM RaST compared to [^18^F]FDG and VLA-4-TC-NM (P < 0.0001 and P = 0.003, respectively) after 72 h of treatment; one-way ANOVA and Tukey's multiple comparisons tests were used for the statistical analysis. **(E)** Treatment of MM1.S cells with VLA-4-TC-NM RaST significantly increased the cell death (VLA-4-TC-NM RaST versus untreated: P = 0.0003; VLA-4-TC-NM RaST versus [^18^F]FDG: P = 0.005; VLA-4-TC-NM RaST versus VLA-4-TC-NM: NS). The cells were treated as in **(A)**. After 72 h, the cells were treated with 0.1 µg PI, and the amount of cell-associated PI was determined with Flow Cytometry; one-way ANOVA and Tukey's multiple comparisons tests were used for the statistical analysis. **(F)** dsDNA breaks were significantly more abundant 72 h after VLA-4-TC-NM RaST. The cells were treated as in **(A)**. After 72 h, the cells were stained with anti-γH2AX and DAPI. Flow Cytometry analysis showed an overall increase in dsDNA breaks during the S-phase. Specifically, dsDNA breaks were significantly more frequent during VLA-4-TC-NM RaST treatment compared to VLA-4-TC-NM (P = 0.003), [^18^F]FDG (P = 0.0002), and the untreated cells (P < 0.0001); 2way ANOVA and Tukey's multiple comparisons tests were used for the statistical analysis. **(G)** Western blotting showed increased levels of apoptosis-related proteins 72 h after VLA-4-TC-NM RaST. The cells were treated as in **(A)**. **(H)** Ferroptosis inhibitor Ferrostatin-1 significantly inhibited VLA-4-TC RaST mediated cell death (P = 0.015). MM1.S cells were plated as in **(C)**. Ferrostatin-1 was administered concomitantly with VLA-4-TC RaST and VLA-4 control at the final concentration of 10 µM. Three-way ANOVA and Tukey's multiple comparisons tests were used for statistical analyses.

Overproduction of ROS leads to non-enzymatic peroxidation of lipids in plasma and organelle membranes with polyunsaturated fatty acids (PUFA), such as linoleic acid, being primary targets 76. The downstream products of PUFA peroxidation, mostly 4-hydroxynonenol (4-HNE), malondialdehyde (MDA), and acrolein, form irreversible protein adducts, leading to prolonged oxidative stress and consequent apoptosis 77. While RaST is known to induce ROS, its mechanism of action has not been explored. We first quantified the ROS that RaST produces by measuring the levels of hydroxyl and peroxyl radicals in MM1.S cells with H_2_DCFDA fluorogenic dye 72 h after treatment. We found that RaST induced significantly higher amounts of ROS in cells treated with VLA-4-TC-NM RaST compared to untreated cells (P = 0.01) or the cells treated with either VLA-4-TC-NM (P = 0.01) or [^18^F]FDG (P = 0.03) (Figure 3A). The persistently elevated ROS levels correlated with an increase in hydroperoxidized lipids, which also increased to a lesser extent with VLA-4-TC-NM alone (Figure 3B). Analysis of data obtained from mass spectrometry identified 3β,5α,6β-trihydroxycholestane (triol), 7-ketocholesterol, and malondialdehyde downstream effectors of lipid hydroperoxidation (Figure 3C). Thus, we uncovered with experimental evidence that the hydroperoxidized lipids profile points to a mechanism where lipid hydroperoxidation is a critical upstream event leading to MMC apoptosis of RaST treated cells. The photophysical nature of the treatment via cytotoxic ROS generation complements current combination pharmacologic drugs that utilize other mechanisms of action.

### VLA-4-TC-NM RaST induces apoptosis and DNA double-strand breaks

Enhancement of lipid-protein adducts directly activates caspase-3, leading to apoptosis 78. Therefore, we investigated the state of caspase-3 activation in VLA-4-TC-NM RaST-treated and untreated MM1.S cells. Although [^18^F]FDG and VLA-4-TC-NM increased caspase-3 levels up to two- and five-folds, respectively, RaST induced a ten-fold higher level of the enzyme than the untreated control 72 h post-treatment (Figure 3D). The upregulation of caspase-3 is a reversible process that does not necessarily culminate in apoptosis-mediated cell death. Using the propidium iodide, we found a direct correlation between activated caspase-3 and cell death. VLA-4-TC-NM RaST mediated cell death was significantly higher than either of the controls, [^18^F]FDG (P = 0.01), and VLA-4-TC-NM (P = 0.04) (Figure 3E).

**Figure 4:**
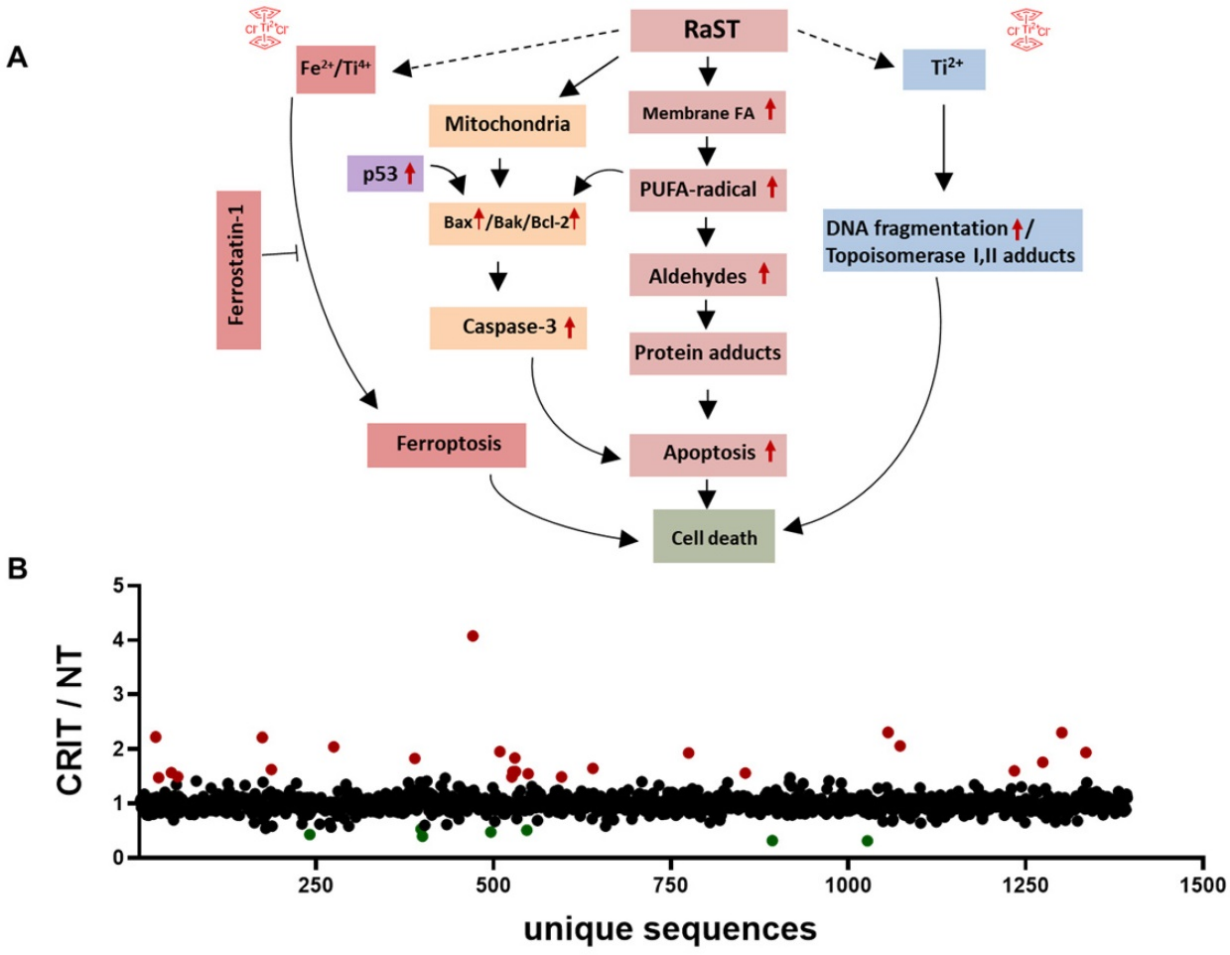
**RaST induces multiple cell death pathways. (A)** Proposed mechanisms of RaST-induced multidimensional cell death pathways. Red arrows are based on data presented in this manuscript. **(B)** Ratios of relative abundances of proteins from either VLA4-TC-NM RaST treated cells or the untreated cells (RaST/NT). Red filled circles: RaST/NT ≥ 1.5; green filled circles: RaST/NT ≤ 0.5. Triplicates of each experimental condition were analyzed.

Protein adducts resulting from hydroperoxidation of PUFA have been shown to interfere with the DNA repair function of p53 77, 79. Therefore, we investigated the downstream effects of p53 mediated DNA repair by measuring the levels of γ-H2A histone family member X (γ-H2AX), a precursor to DNA repair complex assembly and a direct indicator of the DNA double-strand breaks repair activity 71, 80. Our result showed that VLA-4-TC-NM RaST significantly increased the γ-H2AX level compared to controls (Figure 3F). These changes occurred predominantly in the S phase of cell cycle (Figure 3F), suggesting that VLA-4-TC-NM RaST interferes with the cells' ability to synthesize DNA 81. The ensemble of the functional data points to a mechanism where VLA-4-TC-NM RaST initiates lipid peroxidation that leads to apoptosis (Figure 4A) at levels that are significantly higher than those generated by either [^18^F]FDG or VLA-4-TC-NMs alone.

### VLA-4 RaST Upregulates intracellular pro-Apoptotic mediators of cell death

Oxidative stress exerted on a cell induces a number of pro- and anti-apoptotic events, including the upregulation of p53, Bcl-2 antagonist/killer (BAK), Bcl-2-associated X protein (BAX) and Bcl-2 proteins 82-84. To investigate whether RaST modulates the expression of these proteins, we treated MM1.S cells by RaST, [^18^F]FDG or NMs alone for 72 h and analyzed the data relative to the untreated control. The individual proteins were then resolved with an SDS-PAGE and identified by Western blotting with appropriate antibodies. Our data showed that RaST upregulated the expression of BAX, Bcl-2, and p53, but not BAK (Figure 3G), suggesting that BAK protein, which is generally associated with the Bcl-2 family of proteins 85, was not involved in RaST mediated cell death (Figure 4A). These results highlight a RaST-induced apoptosis mechanism in MM1.S cells via BAK-independent Bcl-2/BAX axis.

### Ferroptosis inhibitor abrogates RaST effect

Ferroptosis is a distinct form of programmed cell death that emanates from iron-dependent lipid peroxidation 86, 87. Fenton reactions involving Fe^2+^ ions from the intracellular labile iron pools and peroxides are major contributors to intracellular lipid peroxidation under physiological conditions. Previous studies provided compelling evidence regarding the biochemical similarities of Ti(IV) and Fe(III) 88. During RaST, TC produces Ti-centered radicals that could induce ferroptosis. Therefore, we used Ferrostatin-1 (FST1), an inhibitor of ferroptosis 89, 90, to investigate the contribution of this cell death mechanism to RaST. Whereas RaST inhibited cancer proliferation by 50%, the addition of FST1 restored cell viability, providing evidence for the role of ferroptosis in RaST-mediated cell death (P = 0.01; Figure 3H).

### RaST upregulates pro-apoptotic and downregulates pro-survival proteins

RaST has the potential to perturb multiple pathways associated with cell death. To assess the dynamic changes, we performed label-free MS proteomics with lysates from MM1.S cells treated with VLA4-TC-NM RaST and the untreated controls. Using RaST cutoff ratios of ≥ 1.5 (upregulated) and ≤ 0.5 (downregulated) for the treated to untreated cell lysate (RaST/NT), we identified 23 upregulated and 7 downregulated proteins (Figure 4B, Supplementary Table 1). Among the upregulated proteins, we identified IRF2BPL gene product (RaST/NT = 2.22) and ribosomal protein S15 (RaST/NT = 4.08), which are involved in the cellular response to genotoxic stress through the p53 related mechanism. Apolipoprotein A1 (RaST/NT = 2.31) is a component of PUFA metabolism. In addition to p53 and PUFA pathways, serine hydrolase dipeptidyl peptidase (RaST/NT = 2.22) and nucleoporin POM121 (RaST/NT = 1.96) are implicated in apoptotic pathways. The Ubiquitin-proteasome system components, Ubiquitin E2 (RaST/NT = 2.04) and 26S (RaST/NT = 1.6), are involved in regulating proteolysis. The upregulation of multicopper oxidase enzyme F5 (RaST/NT = 1.8), responsible for inhibiting lipid hydroperoxidation and Fenton reaction, illustrates how MM1.S cells mount countermeasures to inhibit the effects of cytotoxic ROS in response to RaST generates. Beside upregulating pro-apoptotic, RaST also downregulated multiple pro-survival molecules such as those involved in mRNA translation (ATXN2; RaST/NT = 1.6), protein-protein interactions (PDLIM3; (RaST/NT = 0.4), and fatty acid biosynthesis (OXSM; (RaST/NT = 0.5).

Overall, these results point to the lipid hydroperoxidation of PUFA by ROS as the event proximal to RaST agents' internalization. The downstream events likely included the generation of α,ß-aldehydes and protein adducts culminating in cell death mediated by mitochondrial and caspase-dependent mechanisms 91-93. The combined suppression of pro-tumor and upregulation of antitumor proteins, as well as ferroptosis, enable RaST to utilize multidimensional cell death mechanisms to destroy cancer cells and overcome ROS-resistance pathways cancer develop in response to treatment.

### Respective expression of VLA-4 and αvß3 in MM and OCs and GLUT1 in both cell types enable orthogonal RaST

**Figure 5:**
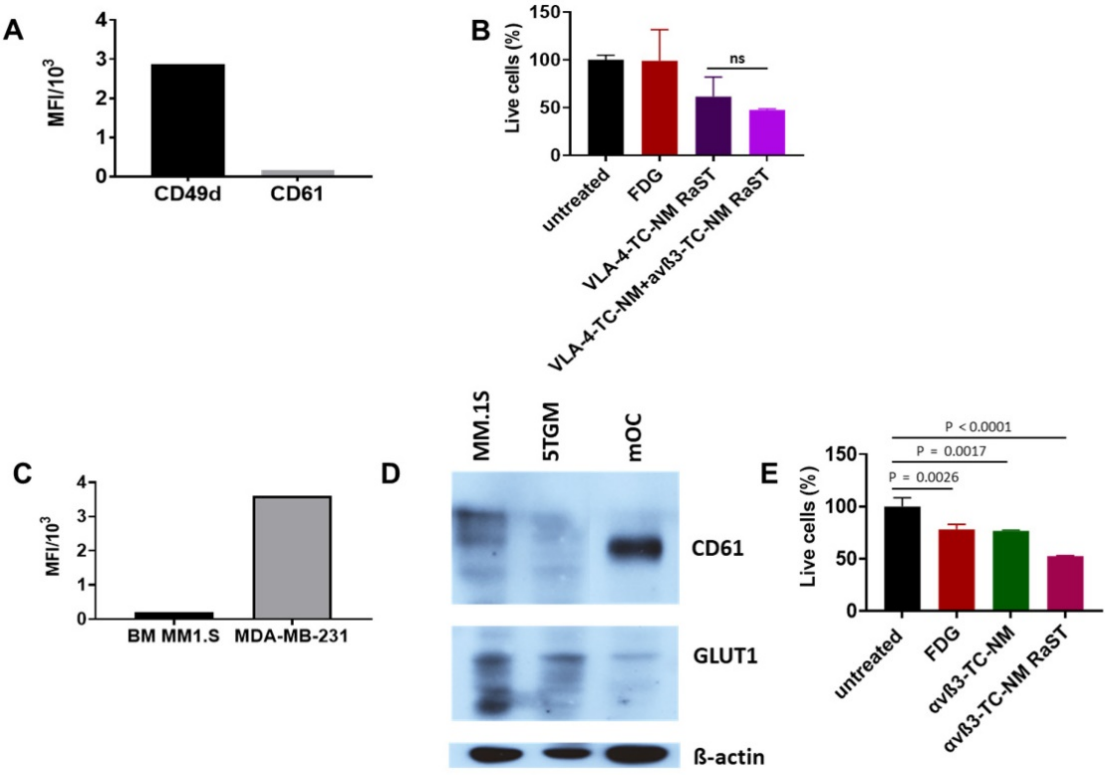
** (A)** CD49d and CD61 expression by MM1.S cells in culture; MM1.S cells were grown in culture with CM for 1-3 weeks prior to flow cytometry analysis. **(B)** VLA-4-TC-NM RaST and VLA-4-TC-NM + αvß3-TC-NM RaST comparison in MM.S1 cells *in vitro*. **(C)** αvß3 expression by MM1.S cells *in vivo* compared to a control. Animals with disseminated MM were euthanized 2-4 weeks after the inoculation and the bone marrow (BM) was examined with flow cytometry for αvß3 expression. MDA-MB-231 human mammary adenocarcinoma cells grown in culture were used as a positive control. **(D)** αvß3 and GLUT1 expression by murine OCs. Bone marrow from femurs and tibias of 5 weeks old C57BL/6-KaLwRij male mice was harvested and differentiated to OCs as described in Shioi, et al. 68. Proteins of interest detected with GLUT1 (clone D3J3A Rabbit mAb) and αvß3 (clone SJ19-09) antibodies. **(E),** αvß3-TC-NM RaST in OC *in vitro.* OCs were differentiated from BMM for 7-14 days prior to the experiment. αvß3-TC-NM RaST was performed as in **(B)**. Averages of each dataset were compared to the untreated control using a one-way ANOVA and Tukey's multiple comparisons test.

Interactions between MMC and OCs determine extensive osteolysis and promote MMC proliferation and resistance to antitumor immunity 94. Inhibition of OC activity with bisphosphonates such as pamidronate and zoledronic acid relies on the high affinity of these drugs for hydroxyapatite in the bone matrix. Although combination therapies that include bisphosphonates have improved skeletal health for MM patients, toxicity of bisphosphonates, incomplete blockade, or reduced availability of bone matrix to bind bisphosphonates (due to osteoblast inhibition) combine to mitigate their routine use in the clinic. The newer and improved denosumab therapy 32 has shown impressive results, with the drawback that precursors can still differentiate once the antibody is cleared. Instead of indiscriminately targeting skeletal bone matrix or inhibiting the function of OCs with different drugs, we explored a treatment paradigm that uses the same drug (TC) and radiopharmaceutical ([^18^F]FDG) RaST to deplete both tumor-associated OCs and disrupt the MM-OC interaction during therapy. This strategy requires orthogonal delivery of TC to OC and MM cells. Unlike metastatic breast cancer to the bone where αvß3 is expressed in both cancer cells and OCs, we found that MM1.S cells were positive for VLA-4 (CD49d), but not αvß3 (CD61) (Figure 5A). Treatment of MM1.S with VLA-4-TC-NM or combined treatment with VLA-4-TC-NM and αvß3-TC-NM, followed by [^18^F]FDG showed that the combination RaST was as effective as the VLA-4 RaST alone (Figure 5B), demonstrating that the low αvß3 expression prevents the contribution of TC-loaded αvß3-targeted NM to RaST effect on MM1.S cells* in vitro*.

Cells exist in 3D *in vivo* via cell-cell interactions that require adhesion molecules. As exposure to the BME can alter integrin expression 46, we determined if the target integrin expression on cancer cells and OCs were retained. After establishing MM1.S in mice, we isolated the bone marrow to measure CD61 expression by flow cytometry. Anti-human CD61 antibody, while efficiently staining the highly ß3-expressing MDA-MB-231 breast cancer cells, was negative for MM1.S, demonstrating that MM1.S express low to no αvß3 in BME *in vivo* (Figure 5C). This finding suggests that αvß3 targeting would be largely ineffective on MM1.S *in vivo*. Expectedly, we found that OCs isolated from murine bone marrow expressed a significantly higher level of αvß3 *in vitro* (Figure 5D), supporting the potential use of αvß3 to target OC for orthogonal strategy. Selective delivery of [^18^F]FDG to target cells requires the expression of GLUT1 on both MM1.S and OCs and cancer cells. Western blot shows that myeloma cell lines MM.1S and 5TGM1 and, to a lesser extent, murine OCs express GLUT1 (Figure 5D). Extension of *in vitro* RaST to TC-loaded αvß3 NM in OCs showed a response similar to the VLA-4-TC-NM RaST in MM1.S cells (Figure 5E), indicating that under similar conditions, both VLA-4 and αvß3 targeted NMs are capable of delivering a sufficient amount of TC into their respective target cells and the GLUT1 level is capable of initiating RaST in both cell types. The differential expression of the target biomarkers allowed us to experimentally dissect the effects of combination RaST on tumor and OC cell populations *in vivo*.

### *In vivo* depletion of OCs potentiates MM response to RaST via orthogonal cellular VLA-4 and αvß3 targeting strategy

**Figure 6:**
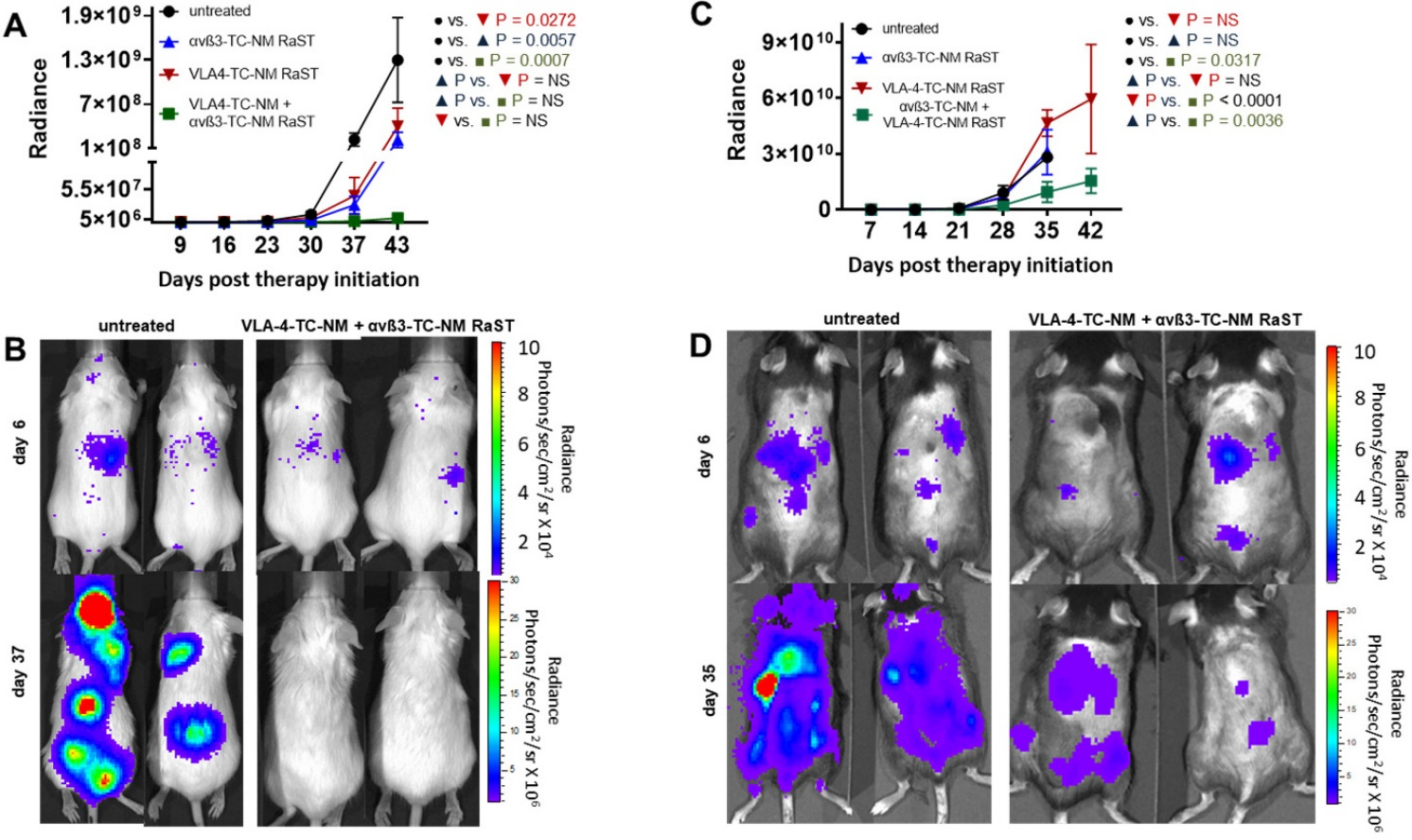
**Dual cancer-osteoclast RaST in mice. (A)** Fox Chase SCID beige MM1.S xenograft animals untreated or treated once weekly for five weeks with αvß3-TC-NM RaST, VLA-4-TC-NM RaST, and αvβ3-TC-NM + VLA-4-TC-NM RaST; MM1.S/CBR/GFP cells (1 x 10^6^ cells per animal) were implanted IV and the therapy was initiated after the whole body radiance reached 1 x 10^6^ photons/sec/cm^2^/steradian (p/s/cm^2^/sr). Tumor progression was monitored with weekly BLI. αvβ3-TC-NM + VLA-4-TC-NM RaST significantly reduced tumor progression compared to no treatment (P = 0.0007).** (B)** Representative bioluminescence images of Fox Chase SCID beige MM1.S xenograft animals treated with αvβ3-TC-NM + VLA-4-TC-NM RaST and the untreated controls. **(C)** C57BL/6-KaLwRij 5TGM isograft animals were implanted with 5TGM/CBR/GFP cells (1 x 10^6^ cells per animal) and monitored as in **(A)**. The test animals were untreated or treated once weekly for five weeks with αvβ3-TC-NM RaST, VLA-4-TC-NM RaST, and αvβ3-TC-NM + VLA-4-TC-NM RaST. αvβ3-TC-NM + VLA-4-TC-NM RaST significantly inhibited tumor progression compared to no treatment (P = 0.0317). **(D)** Representative bioluminescence images of C57BL/6-KaLwRij 5TGM isograft animals treated with αvβ3-TC-NM + VLA-4-TC-NM RaST and the untreated controls. Two-way ANOVA and Tukey's multiple comparisons were used for the statistical analysis.

To demonstrate the application of the combined therapy *in vivo*, we used two animal models of MM. A previous study showed that RaST was able to inhibit MM1.S proliferation and improved survival in an immunocompromised model, although the tumors relapsed due to minimal residual disease that did not respond to treatment 43. In this study, we assessed whether the inactivation of OCs would enhance RaST effect. MM1.S/CBR/GFP cells were injected intravenously into Fox Chase SCID beige mouse strain. The animals were monitored with BLI to detect the appearance of skeletal tumor lesions. We initiated treatments when the total photon flux from the tumors reached 1 × 10^6^ and continued once weekly for five weeks. The animals were imaged once a week and the total photon flux representing the viable MM1.S cells was recorded. Data analysis showed that VLA-4-TC-NM RaST alone produced initial tumor inhibition, which was followed by an increase in tumor growth (Figure 6A, B) similar to what was reported previously 43. Surprisingly, αvß3-TC-NM RaST also suppressed tumor growth compared to the untreated control, with P = 0.006. Given that MM1.S cells do not express αvß3, our result provides new evidence that depletion of OC could disrupt MM-OC interactions or communication networks involved in promoting MMC survival 17, 19, 52. The unique advantage of the orthogonal multicellular over monocellular therapies was evident with the combination αvß3-TC-NM + VLA-4-TC-NM RaST, resulting in unprecedented progression-free survival (P = 0.0007) for this MM model.

Next, we evaluated if the bimodal RaST will improve MM treatment response in an immunocompetent syngeneic murine model for MM by inoculating 5TGM1/CBR/GFP (1 x 10^6^ per mouse) cells in C57BL/6-KaLwRij mice 95. All animals were monitored with BLI and treated as described above. Whereas mice treated with αvß3-TC-NM RaST or VLA-4-TC-NM RaST alone did not exhibit statistically significant tumor response compared to the untreated animals, the combination αvß3-TC-NM + VLA-4-TC-NM RaST significantly inhibited tumor growth (Figure 6C, D). Surprisingly, enhanced tumor proliferation was observed in the VLA-4-TC-NM RaST only mice after day 28 (Figure 6C). As previously reported, immune (T and NK) cells, which are absent in Fox Chase SCID beige, have high VLA-4 expression 96, 97. A likely scenario is that VLA-4 NM distributed TC to MM and immune cells, depending on the relative expression levels of the targeted biomarker. Under this condition, the net amount of TC available to RaST is below the threshold required to exert a therapeutic effect. Consistent with this observation, the correlative increase, albeit gradual, of tumor growth in the combination therapy group could be attributed to the diminishing effect of VLA-4 delivered TC in MMC. Similarly, immune cells also express αvß3 98, which can account for the moderate initial response in the αvß3-TC-NM RaST treated mice before following the trajectory of the untreated mice. Regardless of the relatively poor response using the single RaST approach, the sustainable αvß3-TC-NM + VLA-4-TC-NM RaST response suggests that targeting cancer and multiple cells in BME could have a synergistic effect in cancer therapy.

These data point to a strategy wherein sufficient doses of TC could be delivered locally in the bone to MM, OC, and select immune cells for RaST-mediated simultaneous depletion to achieve complete treatment response. The impressive result from combination RaST in immunocompetent mice suggests that using targetable biomarkers that activate antitumor immune cells could improve progression-free survival in immunocompetent mouse models. As novel combination drug therapies are in high demand for MM, RaST elicits diverse cell death pathways that will complete the standard of care MM therapies.

**Figure 7:**
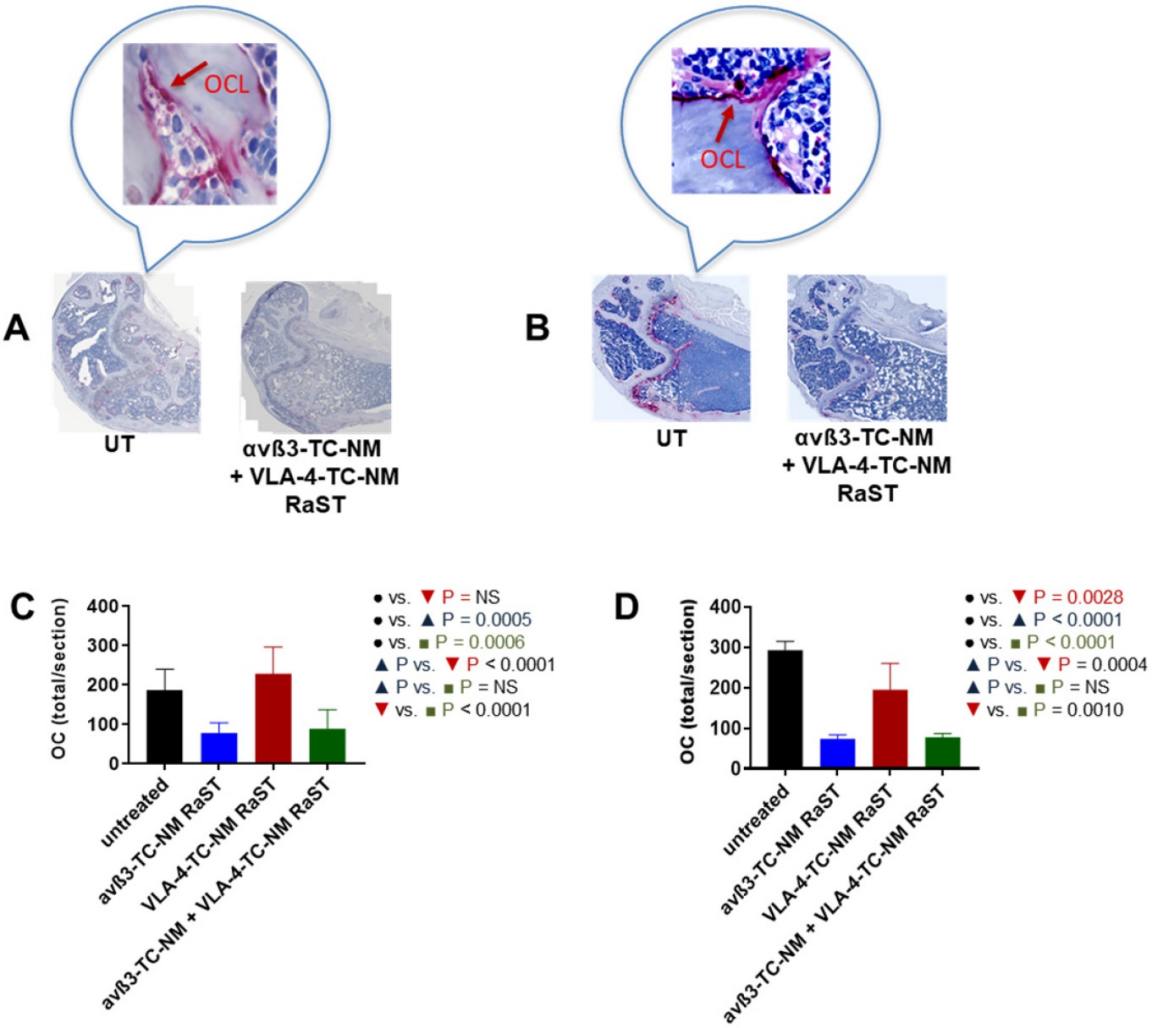
** αvß3-TC-NM RaST leads to OC depletion. (A)** OCs in femurs of Fox Chase SCID beige MM1.S xenograft and **B**, C57BL/6-KaLwRij 5TGM isograft treated with indicated RaST and visualized with TRAP immunohistochemical stain. OC content in **(C)**, Fox Chase SCID beige MM1.S xenograft and **(D)**, C57BL/6-KaLwRij 5TGM isograft was quantified as the total number of TRAP-positive cells per section using Zeiss ZEN 2.3 software. In both models, αvß3-TC-NM RaST, either alone or in combination with VLA-4-TC-NM, significantly reduced the OC population (Fox Chase SCID beige MM1.S xenograft: *P* = 0.0005 and *P* = 0.0006, respectively; C57BL/6-KaLwRij 5TGM isograft: *P* < 0.0001 and *P* < 0.0001, respectively). Two-way ANOVA and Tukey's multiple comparisons were used for statistical analysis.

An important assumption in this study is that αvß3-TC-NM RaST will deplete OCs *in vivo*. Therefore, we investigated the status of these cells in RaST-treated mice with tartrate-resistant acid phosphatase (TRAP), which is widely used as an accurate marker of OC function and population *in vivo* 58, 99, 100. The relative abundance of OC was assessed by quantifying TRAP-positive cells in the bones of untreated and RaST treated (αvß3, VLA-4, or αvß3 + VLA-4 TC loaded NMs) mice using either the MM1.S or 5TGM models as described above. RaST was administered three times, followed by harvesting of femurs for TRAP immunohistochemistry. TRAP-stained OCs were visible on the bone surface of untreated mice (Figure 7A,B). Analysis of TRAP-positive cells revealed that αvß3-TC-NM and αvß3-TC-NM + VLA-4-TC-NM RaSTs were equally effective in eliminating OC *in vivo* in the MM1.S xenograft (Figure 7A) and 5TGM isograft (Figure 7B) models, as revealed by the reduced or discontinuous TRAP-stain. Quantification of the data showed that 5TGM MM exhibited about a 3-fold decrease in OC relative to controls (Figure 7D), but a marginal reduction in the OC population was observed for VLA-4-TC-NM RaST. A similar trend was found in the immunocompromised mouse model, with about a two-fold decrease of OCs in αvß3-TC-NM RaST from control (Figure 7C). Expectedly, the combination αvß3-TC-NM + VLA-4-TC-NM RaST exhibited a similar profile as αvß3-TC-NM RaST alone. These observations confirm that αvß3-TC-NM RaST is primarily responsible for inhibiting osteoclastogenesis and combines with VLA-4-TC-NM RaST to potentiate MM treatment response.

## Discussion

Traditional combination chemotherapies typically use two or more pharmacologic agents to sustain therapeutic effects in cancer cells 101, but these drugs also introduce cumulative side effects. Particularly, MM is a fatal malignancy that requires a multidrug therapeutic regimen to overcome non-responsiveness and resistance. However, end-organ damage, age, and co-morbidities render MM patients fragile, further preventing aggressive treatments due to off-target toxicities. To minimize systemic and organ toxicities, we pioneered a unique treatment paradigm, RaST, which is based on the orthogonal delivery of individually nontoxic amounts of a light-sensitive drug and a radiopharmaceutical to cancer cells where they converge to produce toxic amounts of ROS 42, 43. RaST has the unique advantage of using a photophysical ROS-generating mechanism to eradicate target cells, regardless of their origin. This scenario allowed us to use a single photosensitizer (TC) instead of multiple drugs for simultaneous MM-stromal cell therapy. Our data highlights multiple cell death pathways involved in TC-mediated RaST. First, RaST stimulates lipid hydroperoxidation of PUFA 77, 102-105, a process that produces various α,ß-aldehydes known to form irreversible function-perturbing protein adducts and culminates in caspase-3 independent apoptosis induction (Figure 4A). Second, RaST also utilizes a caspase-3 dependent apoptosis pathway via Bax-independent Bak/Bcl-2 axis. Both of these apoptosis-mediated cell death pathways emanate from ROS generation. Third, VLA-4-TC NMs induce double-stranded DNA breaks by a ROS-independent pathway 106 that relies on stable titanium-centered radicals to produce DNA damage 42. While this mechanism may have limited contribution to cell death due to the small doses used, it reveals another path that could create a multidimensional combination therapy that uses TC as both a photosensitizer and a pharmacologic drug at higher doses. Finally, the surprising observation of ferroptosis as a contributor to RaST-mediated cell death points to a mechanism in which the biochemical similarity of iron and titanium ions 88 enhance the Fenton reaction during TC-mediated RaST. Based on the above processes, the observed delay in the effective killing of MM cells following RaST was not surprising as these cell death pathways are not instantaneous. During this progressive cell death process, it is possible to amplify the treatment response by adding specific pharmacological drugs that leverage any of the above pathways to minimize residual disease.

Our study reveals the effectiveness of orthogonally targeting RaST against cancer cells and their microenvironment in both immunocompetent and immunocompromised MM mouse models. While VLA-4 RaST primarily accounted for anti-myeloma activity *in vitro*, both αvß3 and VLA-4 RaST individually exerted a therapeutic effect on MM in both models. The data illustrates how disruption of cancer-bone cell communication network alone using the non-MM targeting αvß3-TC-NM could inhibit cancer proliferation. Other studies have shown that treatment of MM with the anti-resorptive bone agent, zoledronic acid 107, and the Receptor Activator of NF-kB ligand (RANKL) inhibitor, denosumab 32 reduced osteolysis and inhibited MM progression 108. Unfortunately, renal clearance of bisphosphonates has often raised concerns for their tolerability in MM 32, and rebound effects due to activation of stalled OC precursors upon discontinuation of denosumab are an emerging weakness of antibody treatment 109. Although our single cell-targeted RaST does not have similar limitations, all treated animals experienced MM rebound after a latent period, similar to what is found in standard therapies. Implementation of the MM-OC combination RaST using αvß3-TC-NM + VLA-4-TC- NM *in vivo* achieved prolonged progression-free survival for these mice. These data provide evidence that the dual targeting of cancer cells and their microenvironment can reduce both osteolysis and MM tumor burden *in vivo* by depleting OCs within the tumor region.

We found stark differences in treatment response between the immunocompetent and immunocompromised MM models. Not only did VLA-4 RaST have a negligible effect on tumor progression compared to the untreated control, but the tumor cells in treated mice also appeared to proliferate at a faster rate than control in the immunocompetent model. At the low dose of TC used in this study, the presence of VLA-4 expressing immune cells in this model could reduce the effective dose of TC available for effective RaST in MM cells, which was not the case in the immunocompromised model. Similarly, the αvß3 RaST followed a similar trajectory as the untreated control, albeit with low tumor inhibition at the early stages. Histological assessment of bone tissue in both mouse models reveals that at basal levels, the OC population was higher in C57BL/6-KaLwRij strain relative to the Fox Chase SCID beige strain. Logically, this imposes a higher threshold of OC depletion in the immunocompetent model to achieve a therapeutic effect, which accounts in part for the limited inhibition effect on MM. The fact that the combination of two apparently ineffective VLA-4 and αvß3 RaST inhibited tumor growth *in vivo* supports a treatment paradigm where the perturbation of communication between cancer and multiple enabling cells can synergize to exert durable tumor inhibition.

Collectively, this study elucidates the multidimensional cell death mechanism that RaST uses to exert a therapeutic effect. It also uncovers an orthogonal multicellular treatment paradigm targeting both cancer cells and their microenvironment by activating the same drug type to enhance cancer therapy. By using a photophysical method to produce cytotoxic radicals in target cells, the dual cell targeting RaST strategy can add a new dimension to current combination therapies that only use pharmacologic drugs. In particular, the latent period between therapy initiation and the onset of cell death (3 days) provides a therapeutic window when cancer cells are most vulnerable to additional assaults. This timing could be utilized to eradicate residual disease before it transforms into a therapy-resistant phenotype.

## Supplementary Material

Supplementary table.

## Abbreviations

BM: bone marrow
BME: bone microenvironment
BMSC: bone marrow stromal cells
ECM: extracellular matrix proteins
[^18^F]FDG: ^18^F-fluorodeoxyglucose
FST1: ferrostatin-1
4-HNE: 4-hydroxynonenol
MDA: malondialdehyde
MGU: monoclonal gammopathy of undetermined significance
MM: multiple myeloma
MMC: multiple myeloma cells
OC: osteoclast
RaST: radionuclide stimulated therapy
ROS: reactive oxygen species
SMM: smoldering multiple myeloma
TC: titanocene
VLA-4: very late antigen-4
