# Supplementary material for: Orthogonal targeting of osteoclasts and myeloma cells for radionuclide stimulated dynamic therapy induces multidimensional cell death pathways
Source: Theranostics. 2021 Jun 22;11(16):7735–54. doi: 10.7150/thno.60757 (PMC8315072; doi:10.7150/thno.60757)
Supplement: Supplementary file 1 — Supplementary table. [file thnov11p7735s1.pdf]

Supplementary Table 1. Annotated label-free proteomics analysis of MM.1S cells that were treated with VLA-4 CRIT and the untreated controls. The samples were analyzed in triplicate, and the ratios of CRIT treated cells and untreated cells were obtained.

GENE	FOLD OVER UNTREATED	PUTATIVE FUNCTION
DPP7	**2.22**	Metabolic serine hydrolase/dipeptidyl peptidase implicated in apoptosis and glucose homeostasis
IRF2BPL	2.22	Transcription factor targeted by p53 in response to genotoxic stress
UBA2	2.04	ThiF domain implicated in binding to Ubiquitin E1/2
RPS15	4.08	Ribosomal protein S15 implicated in p53 function
LOC441081	1.96	Sequence of nucleoporin POM121 implicated in regulation of apoptosis
APOA1	2.31	Apolipoprotein A1 implicated in PUFA metabolism
KIAA0196, WASHC5	2.06	Strumpellin implicated in actin mediated intracellular vesicle transport
KRT9	2.3	Type 1 intermediate filament implicated in intracellular vesicle transport
HBA1-2	1.5	Hemoglobin subunit alpha implicated in intracellular antioxidant mechanisms
PSMD8	1.6	26S proteasome component involved in regulated proteolysis
DCTN2	1.5	Dynactin subunit 2 involved in microtubule mediated intracellular transport
DDX18	1.6	ATP-dependent RNA helicase involved in nucleic acid replication
KRT1	1.8	Keratin 1, component of intermediate filament system
WDR43	1.5	WD40 repeat, component of rigid protein scaffold system implicated in metabolic response to stress
SRP72	1.6	Signal recognition particle subunit involved in protein transport
F5	1.8	Multicopper oxidase enzyme involved in electron transport and oxidation of various compounds
CASKIN1	1.6	SH3-like protein binding domain involved in signal transduction and response to stress

SEH1L	1.5	Nucleoporin SEH1 involved in mTOR signaling pathway and RNA transport
HLA-DRA	1.7	HLA class II histocompatibility antigen, DR alpha chain, involved in PD-1 signaling
DHX30	1.9	DEAD/DEAH box helicase involved in cell organization and biogenesis, including response to stress
Sec16A	1.6	Protein transport protein Sec16A
AGMAT	1.6	Agmatinase, mitochondria localized enzyme thought to be involved in response to stress
SSSCA1	1.8	Sjoegren syndrome/scleroderma autoantigen 1, a Zn ribbon domain 2 protein with unclear function
FBL	1.9	rRNA 2'-O-methyltransferase fibrillarin thought to be envolved in repair of non-enzymatically damaged proteins
C7	0.4	Conserved protein sequence with no known function
MTDH	0.5	Protein LYRIC, involved in cell organization and biogenesis; regulation of biological process; response to stimulus
PDLIM3	0.4	LIM domain consisting of two Zn fingers thought to be involved in mediating protein – protein interactions
CCDC86	0.5	Coiled-coil domain-containing protein 86 induced by IL3 and upregulated in multiple cancer types
OXSM	0.5	Beta-ketoacyl-ACP synthase involved in fatty acid biosynthesis
GOLPH3	0.3	Golgi phosphoprotein 3 involved in regulation of Golgi trafficking
ATXN2	0.3	Ataxin-2 involved in regulation of mRNA translation
